# Improved accuracy in automated chemistry through the use of reference materials

**DOI:** 10.1155/S1463924680000545

**Published:** 1980

**Authors:** R. F. Coleman

**Affiliations:** National Physical Laboratory Teddington Middlesex UK


### Improved accuracy in automated chemistry through the use of reference materials*

**R. F. Coleman**
*National Physical Laboratory, Teddington, Middlesex, UK.*

### Introduction

The development of automated analysis has arisen from the need for increased productivity because of the growing number of measurements required for scientific, commercial and legislative purposes. Many manufacturers have responded to this challenge and developed all kinds of instruments for chemical analysis and for measurement of physical and mechanical properties. Naturally, the main emphasis has been on minimising the capital cost of the instrument and increasing the throughput of samples so that the overall sample costs are small. Rarely has the accuracy of measurement been given the weight it deserves. Interlaboratory studies frequently show that samples measured by one instrument give quite different answers on another. This is clearly unsatisfactory because unless there is adequate compatibility of measurement, communication is inhibited and indeed the actual analysis may be virtually worthless.

In many commercial matters, such as the buying and selling of raw materials, a contract exists specifying quality and quantity of material, and can quite easily include methods of analysis so that the buyer and seller both use the same method and therefore can transfer information easily. Whether the measurement is accurate or close to the true value is of little significance. If more than two laboratories are involved, frequently the solution is to try to harmonise measurements through a collaborative exercise involving analysis of samples from a single homogenized batch. It is a common experience for the results to show a wide variation in the first instance indicating serious discrepancies in the performance of individual laboratories. Through discussion and elimination of some of the worst methods/laboratories, it is often possible to come to a consensus and the participating laboratories can yield subsequently consistent results. Unfortunately, with time this consistency may be reduced and also it is very difficult for outsiders to enter the measurement group without all participants going through another collaborative exercise.

Another method often favoured by legislators to reduce laboratory variation is to fix on a particular technique and insist that it is used by all laboratories for all time. In such cases it is often necessary to choose a labour intensive method because it is only in this way the small laboratories can participate, but this automatically inhibits many laboratories from the use of fast automated techniques and causes problems if there are a large number of samples to be measured. As innovators, the developers of automated methods would obviously find prescribed methods a disadvantage. In order to minimise this problem they have a prime responsibility to demonstrate that their techniques not only provide faster and more economic solutions, but also they do not introduce confusion and incompatibility into the measurement system.

In the past some automated instruments have appeared on the market which were quite obviously incompatible because they were not measuring the same analyte as the routine manual method. For example, the measurement of glucose in blood plasma was changed by the automated method to the measurement of reducing sugars, and clearly these cannot be compatible if a less specific method is used. In the current instruments used for the detection of the adulteration of milk by water, thermistors have replaced calibrated Hortvet thermometers in order to increase their speed of operation but at the expense of the introduction of unquantified systematic errors because of the lack of traceability to national standards for temperature.

At the present time in Western Europe nearly all our measurement systems are voluntary but it is evident that this has caused problems and gradually, prescribed methods or methods which have traceability to national laboratories are becoming mandatory in specific areas. In the environmental field in the United States, all measurements on pollutants in car exhausts must be traceable through reference materials back to the National Bureau of Standards (NBS). This applies not only to measurements made in the United States, but foreign manufacturers wishing to export cars to the United States must also use prescribed methods and traceability to NBS. Unless manufacturers and users take note of these trends and make voluntary changes it is likely that considerable pressure will be applied in the future, and perhaps legislation, to ensure compatibility of measurement.

### Compatibility through accuracy

There are various ways in which compatibility can be achieved. It is suggested that the best means for clinical and industrial purposes, is that the data obtained should be accurate within the limits of uncertainty required by the end use. For this discussion we will define an accurate measurement as being both precise and free from systematic error so that accurate measurements are therefore necessarily compatible. Thus we would ask that the developers of automated instruments should not only seek rapid operation at low cost but should also include accuracy and if possible wide dynamic range as important requirements of any new instrument.

There are several different ways of achieving compatibility and transferring accuracy between 2 or more laboratories. The most common of these are

*a) Calibration services*

A central standards laboratory may calibrate instruments and other artefacts sent to it by laboratories in the measurement network. The calibrated instruments are returned for use as secondary standards and then can be used to transfer accuracy to field methods often at remote installations. This is the method widely used for transferring the accuracy of physical measurement parameters such as length, mass, voltage, etc. In general it is not used for instruments measuring chemical or materials properties.

**Paper presented at the conference "Automation in Industrial and Clinical Chemistry", City University London, July 1979.*

*b) Standard reference data*

The use of critically evaluated data on the properties of well characterised materials can form a basis for calibration. For example, the refractive index and density of water are accurately known and, provided pure water is available, an instrument manufacturer can calibrate his own equipment using the appropriate reference data.

*c) Reference materials*

Probably the best way of transferring accuracy when automated instruments are used is through reference materials. This is a device or physical system to which definitive numerical values can be applied to specific properties. In this sense a reference material could be a set of weights used for mass measurements or a chemical system that emits an accurate and reproducible amount of substance, eg gas generators or a homogeneous stable alloy containing a known amount of a particular element. It is through the use of reference materials that it is possible to assess the whole of the measurement process.

*d) Reference methods*

Reference methods provide another means of achieving compatibility but it has been found that particularly good protocols must be written and tentative reference methods require extensive testing in several laboratories before they are in a state suitable for widespread use. Frequently reference methods and materials are used together.

### Systems approach to measurement compatibility

The National Bureau of Standards in the United States has proposed a systems approach to measurement compatibility and recognises that certified reference materials provide a necessary but by no means sufficient mechanism for achieving measurement compatibility on a national or international scale. The systems approach is illustrated in Figure 1, and described by Uriano and Gravatt [1]. Through a hierarchical system of analytical methods and reference materials it is possible to transfer accuracy from a single national laboratory throughout a large network of laboratories. As one proceeds from basic measurement units to field methods the accuracy requirements diminish at the expense of increased measurement efficiency. In such a system a typical field method accuracy of 5–10% would require the accuracy of the reference method to be in the range of 1–3% and probably the definitive method and primary reference material should have accuracies of better than 1%.

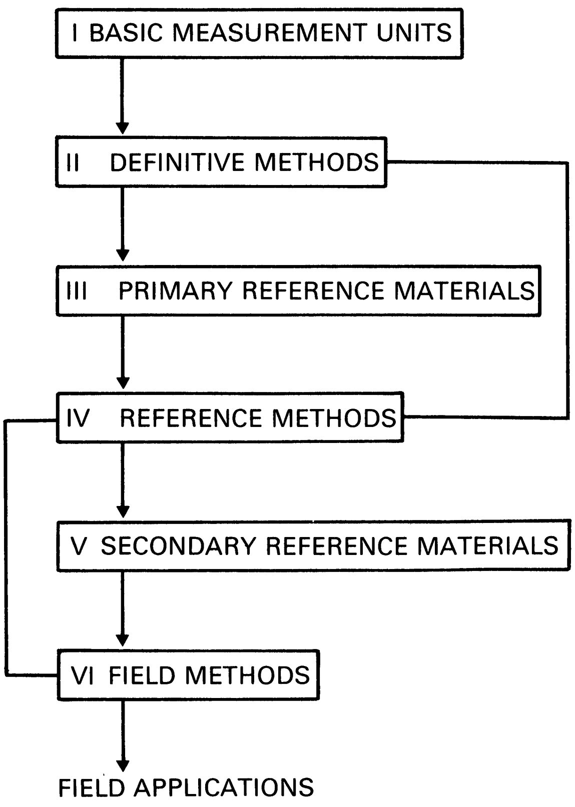

*Figure 1. Six basic technical components of an idealised accuracy-based compatible measurement system.*

It is not my intention to suggest that all reference materials should be produced by national laboratories such as UK NPL or US NBS. There is a need for other organisations to participate because the variety and quantity of reference materials required by method development and particularly for quality control is large. It is important however that if compatibility is to be achieved nationally and eventually internationally, secondary and tertiary reference materials are traceable through a defined protocol back to reference methods (precise and free from bias) and primary certified reference materials.

Some practitioners have resisted this approach and claim that precision was all that was needed to achieve compatibility. It can be shown that this is satisfactory for a limited number of laboratories that are in close contact and frequently exchange samples for measurement. Unfortunately, this makes it difficult for outsiders to achieve compatibility with those inside the select group and also does not take care of the variations in laboratories over time.

### Criteria for the development of reference materials

The first consideration should be to assess the technical requirements which would dictate the end use for the reference material. This will largely govern the important certification requirements such as accuracy, stability and physical form of the material.

Prior to the development of reference materials it is also necessary to develop an adequate means of measuring the property and preferably one which is linked to basic measurement standards such as those of length, time, current etc.

A range of other properties need to be considered, particularly stability and homogeneity. If the material is to have a long term use then clearly stability is important and much of the cost of developing reference materials is due to extensive testing for stability. The end use requirements will dictate the physical form and it is important that the reference material is available in a form which can be directly applied to the instrument being calibrated. In clinical chemistry pure compounds such as glucose, cholesterol etc, have been available for some time, but an automated instrument determining these parameters in serum requires reference materials in which these analytes are present in serum in the usual physiological range of normal concentrations. Any other form will introduce errors into the system with the present generation of analytical instruments for clinical chemistry. The tendency has been to develop instruments which will undertake very specific tasks and this in turn requires specific reference materials for calibration. For the future it would be much better if the next generation of instruments were more flexible and could for example analyse serum and water directly in the same instrument and thus limit the range of reference materials required for calibration purposes.

I am often surprised to find how many laboratory workers are unfamiliar with the range of reference materials already available at relatively modest cost. National organisations such as the US National Bureau of Standards (NBS), the UK National Physical Laboratory (NPL) and Bundesanstalt fur Materialprufung (BAM) have been providing certified reference materials for many years and commercial organisations such as the Bureau of Analysed Samples also provide certified materials in specific forms. In 1980 we can expect to see an International Standards Organisation publication which is a directory of the world's certified reference material suppliers. Details of the CRM's available from each manufacturer may then be obtained by writing directly and asking for brochures. In the longer term this information may be available from a single central source. A list of UK suppliers with addresses for the major categories of CRM's listed in Table 1 is now available as an NPL report [2].

### Examples where the need for compatibility is apparent

### Haemocytology

Throughout the world several billion blood samples are analysed each year for blood count and particle size. However, despite this large number of analyses the measurements from one laboratory are frequently incompatible with those from other laboratories. Palliative measures have been taken by some manufacturers who supply secondary reference samples to their customers to try and achieve compatibility of measurements from laboratories using instruments of a specific type. Unfortunately, secondary standards are unstable, with a life of only a few weeks. Currently a scheme (Figure 2) has been proposed by NPL, in conjunction with the International Committee for Standardisation in Haemocytology, which envisages the production and characterisation of a number of primary stable reference materials in the relevant size range. These primary reference materials would be used to calibrate the particle size scales of primary blood counting instruments maintained at NPL. These calibrated reference instruments would in turn be used to certify batches of stabilised blood which would then serve to transfer the calibration to reference instruments maintained by manufacturers and national and regional quality control services. In the longer term it is hoped that the primary reference materials will be characterised by intercomparison between expert laboratories throughout Europe and hence achieve compatibility on an international scale. A feasibility study is also proposed for the production of blood-like oblate spheroidal polymer particles which would model erythrocytes and platelets in respect to size, shape and rheology. If it is shown that these can be produced in the longer term the "stabilised" blood which currently lasts a few weeks could be replaced by surrogate reference materials which mimic blood in all properties required by the blood counting and size instruments. In my view this kind of activity is an example in which instrument manufacturers, calibrating laboratories and regulatory agencies need to collaborate as there are substantial benefits for all if we can achieve compatibility.

**Table 1. Major categories of CRM's now available**

| | |
|---|---|
| 1 | Geological (ores, minerals, etc) |
| 2 | Physico-chemical |
| 3 | Nuclear and radioactivity |
| 4 | Environmental (air, water, particulates) |
| 5 | Ferrous metals |
| 6 | Non-ferrous metals |
| 7 | Chemicals, inorganic |
| 8 | Chemicals, organic, including petroleum |
| 9 | Paper |
| 10 | Plastic, rubbers, plastomers |
| 11 | Glasses, ceramics, refractory materials |
| 12 | Biological, botanical, including foods |
| 13 | Pharmaceuticals |
| 14 | Clinical chemistry applications |
| 15 | Technological or engineering |

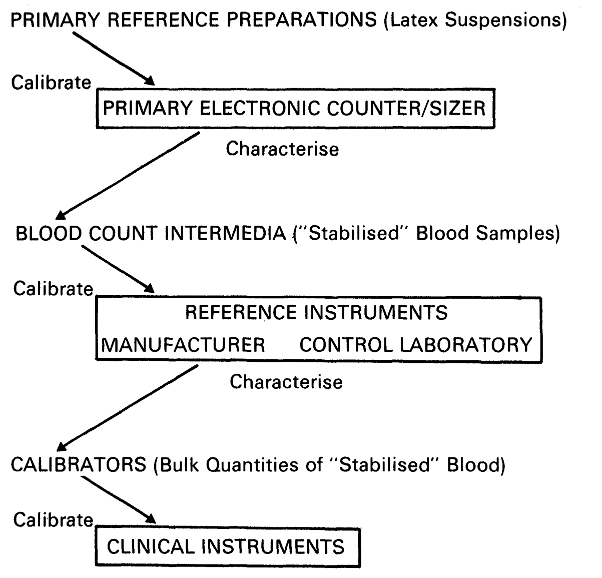

*Figure 2. The ICSH scheme to promote compatibility of measurement in haemocytology.*

### Serum analysis

For 20 years, automation or, more correctly, mechanisation has dominated the clinical biochemistry laboratories' approach to its routine measurement workload. In the transition from manual to automated methods, repeatability has commonly displaced accuracy as the main requirement for valid results. It is now widely but not universally accepted that the arbitrariness of calibrating multi-channel systems with materials having manufacturers assigned values is undesirable. This is one reason why the US National Committee for Clinical Laboratory Standards is promulgating recommendations as to suitable criteria for calibration reference materials and the correct methodological principles for establishing assigned values.

The excellent precision of modern analysers allows data to be accumulated on inter- and intra-individual variations in blood biochemistry and their true dependence. It is, however, impossible to relate data extended over time and space – from one laboratory to another or to compare with published values – without building accuracy into the methodology.

Substantial progress is being made in the development of reference materials and methods for serum analysis. Definitive methods and primary reference materials are available for most important electrolytes and for some of the small organic molecules such as uric acid and cholesterol. Those for glucose, creatinine and urea are not far from completion. A quality control sample for clinical chemists analysed by definitive methods has been circulated in the USA and proposals for similar activities in the UK have been made by NPL. Production of a lyophilised serum certified reference material is in progress at US NBS at present and should be issued and certified for the above constituents in 1980.

A serum reference material for 4 common anti histamine drugs at normal concentrations has recently been issued by US NBS. The EEC, through the Community Reference Bureau (BCR), also expects to certify reference materials relevant to clinical chemists in the near future. This includes serum with 4 hormones, cortisol, oestradiol, progesterone and testosterone at the normal physiological concentrations and a serum CRM with drugs phenytoxin and phenobarbitone at therapeutic and toxic concentrations.

### Conclusions

A need has been indicated for more attention to be given to achieving compatible measurements from automated instruments. It is argued that repeatability is not a sufficient criterion for assessing the reliability of analytical methods and instruments. Co-operation is required between manufacturers, their customers and suppliers of certified reference materials, to ensure the necessary work to achieve compatibility is undertaken in good time to meet the needs at a realistic cost.

ACNOWLEDGEMENTS
I would like to thank my colleagues at the National Physical Laboratory for useful comments on the content of this paper.

REFERENCES

[1] Uriano, G. A. and Gravatt, C. C., CRC Critical Reviews in Analytical Chemistry, October 1977, 361.
[2] Cox, J. D. and Ridsdale, P. D., NPL Report Chem 93, 1978.

### Simple method for calculation of the frequency of standardisation of analytical measurements

**J Inczédy**
*Institute of Analytical Chemistry, University of Chemical Engineering, Veszprém, Hungary.*

### Introduction

In analytical chemical measurements, when measurements are periodically repeated or the measurement itself takes a long time, for example in process control, the most significant source of error is the long term instability of the instrument. In instrumental analytical measurements, the precision of the measurement is often acceptable although the bias can be as much as 20–50% due to the shift of the zero point of the instrument. In many cases, the latter problem is not considered by the analyst because it is one which is not easily grasped.

In this paper a simple method is presented by which the approximate frequency of standardisation of the instrument can be calculated.

Recently, several papers have appeared in the literature dealing with the acceptable total error (that is, both systematic and random errors) of analytical measurements [1 and 2]. Also, a method of eliminating the error caused by the shift of the zero point using a linear interpolation has been described [3].

### Calculation of the time interval between standardisations

For the calculation of the optimum time interval between two standardisations, the following assumptions and considerations are made:-

(i) That there is a steady state zero point migration which can be approximated by a periodic sine wave function.
(ii) That the shift between and during the two standardisation procedures can be approximated with a linear function.
(iii) That the distribution of the results of measurements is of a normal Gaussian form.
(iv) That the standardisation must be repeated when the error arising from the migration becomes significant in relation to the standard deviation of the measurements.

The first assumption was made because the slow migration of the zero point of the instrument in most cases is periodic and the time of the period depends on the periodic changes of the environment or electrical mains load.

The second assumption is also valid since the time interval between the standardisation points is usually much smaller than the time of the period.

In analytical measurements, the third assumption is accepted.

The fourth assumption is made because the difference between a measured value obtained as the mean of close successive measurements and another one will be significant when the limit of 2s is passed. ('s' is the standard deviation of the 'parallel measurements', carried out during a short time interval $\tau$).

Between the two successive standardisations the error caused by the slow migration of the zero point can be taken into consideration when assessing the true measurements by linear interpolation if the migration rate is known. This is possible only in those cases where very rigorous measurements are necessary.

Figure 1 shows a diagram of the zero point migration along with two idealised curves. The original curve can be approximated by a sine wave function or by an equivalent regular triangle function. The sine wave function and the triangle function are equivalent when their period time T and variance $\sigma^2$ are the same.

According to the calculations, the sine and triangle functions exhibit the same variance when their amplitudes are in the following relation:

$$a = 0.8246\ A \qquad (1)$$

a is the amplitude of the sine wave function, and A is the peak amplitude of the triangle function. Their variance may be expressed as follows :

$$\sigma^2 = \frac{a^2}{2} \qquad (2)$$

A small idealised part of Figure 1 is shown in greater detail in Figure 2, where the curve is approximated with a straight line. During and after the setting of the zero point

